# *cis*-Diiodido(*N*,*N*,*N*′,*N*′-tetra­methyl­ethylenediamine-κ^2^ *N*,*N*′)palladium(II)

**DOI:** 10.1107/S1600536812033004

**Published:** 2012-07-28

**Authors:** Antonio Abellán-López, María Teresa Chicote-Olalla, Delia Bautista-Cerezo

**Affiliations:** a Grupo de Química Organometálica, Departamento de Química Inorgánica, Universidad de Murcia, Murcia 30071, Spain; b SAI, Universidad de Murcia, Murcia 30071, Spain

## Abstract

In the title complex, *cis*-[PdI_2_(C_6_H_16_N_2_)], the Pd^II^ atom lies on a crystallographic twofold rotation axis and is four-coordinated by the two N atoms of a chelating *N*,*N*,*N*′,*N*′-tetra­methyl­ethylenediamine ligand [Pd—N = 2.125 (3) Å] and two I atoms [Pd—I = 2.5833 (4) Å], displaying a distorted square-planar geometry (r.m.s. deviation = 0.005 Å), imposed by the small bite of the chelating ligand [N—Pd—N angle = 84.68 (18)°].

## Related literature

For related diiodido complexes, see: Jones *et al.* (2007 ▶); Wursche *et al.* (1999 ▶); Dodd *et al.* (2006 ▶); Alsters *et al.* (1993 ▶); Bhattacharyya *et al.* (2009 ▶); Ha (2009 ▶, 2010 ▶). For mol­ecular parameters in related dichlorido complexes, see: Boyle *et al.* (2004 ▶); Iball *et al.* (1975 ▶). For a description of the Cambridge Structural Database, see: Allen (2002 ▶).
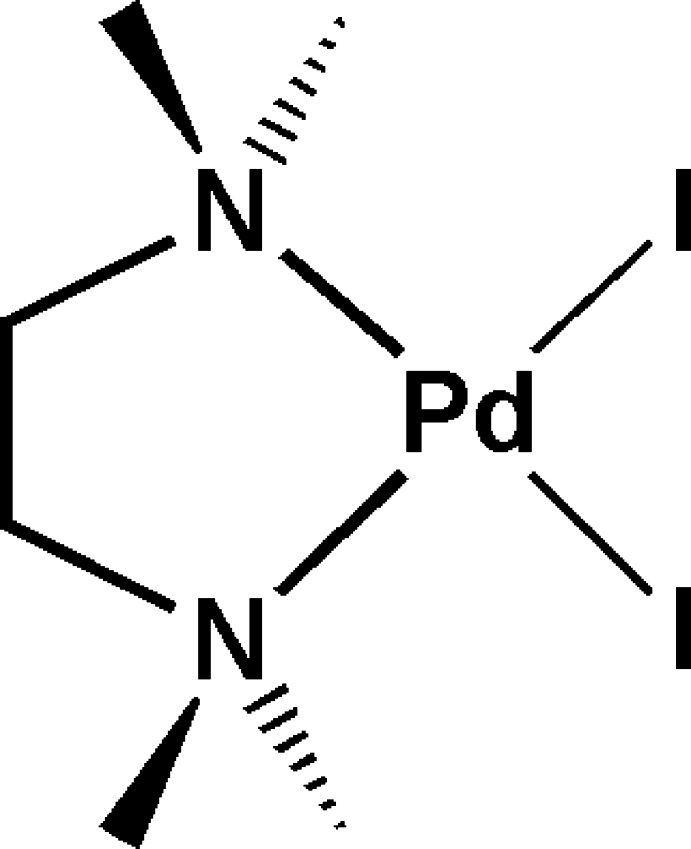


## Experimental

### 

#### Crystal data

[PdI_2_(C_6_H_16_N_2_)]
*M* *_r_* = 476.41
Monoclinic, 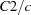

*a* = 7.9266 (4) Å
*b* = 14.6911 (7) Å
*c* = 10.5309 (5) Å
β = 107.262 (2)°
*V* = 1171.09 (10) Å^3^
*Z* = 4
Mo *K*α radiation
μ = 6.81 mm^−1^
*T* = 100 K
0.22 × 0.15 × 0.03 mm

#### Data collection

Bruker SMART APEX CCD diffractometer
Absorption correction: multi-scan (*SADABS*; Bruker, 2003 ▶) *T*_min_ = 0.581, *T*_max_ = 0.822
6502 measured reflections
1351 independent reflections
1326 reflections with *I* > 2σ(*I*)
*R*_int_ = 0.020

#### Refinement

*R*[*F* ^2^ > 2σ(*F* ^2^)] = 0.023
*wR*(*F* ^2^) = 0.060
*S* = 1.14
1351 reflections
53 parameters
H-atom parameters constrained
Δρ_max_ = 1.23 e Å^−3^
Δρ_min_ = −0.73 e Å^−3^

### 

Data collection: *SMART* (Bruker, 2001 ▶); cell refinement: *SAINT* (Bruker, 2003 ▶); data reduction: *SAINT*; program(s) used to solve structure: *SHELXS97* (Sheldrick, 2008 ▶); program(s) used to refine structure: *SHELXL97* (Sheldrick, 2008 ▶); molecular graphics: *SHELXTL* (Sheldrick, 2008 ▶); software used to prepare material for publication: *SHELXTL*.

## Supplementary Material

Crystal structure: contains datablock(s) I, global. DOI: 10.1107/S1600536812033004/lr2073sup1.cif

Structure factors: contains datablock(s) I. DOI: 10.1107/S1600536812033004/lr2073Isup2.hkl

Additional supplementary materials:  crystallographic information; 3D view; checkCIF report

## Figures and Tables

**Fig. 1.**
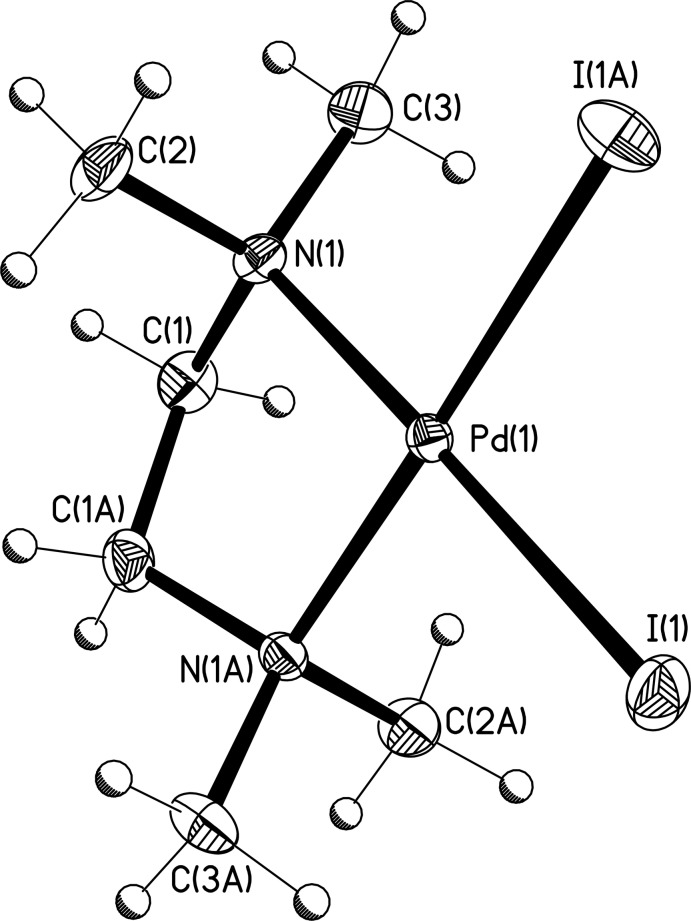
The molecular structure of the title compound. Ellipsoids represent 50% probability levels.

**Fig. 2.**
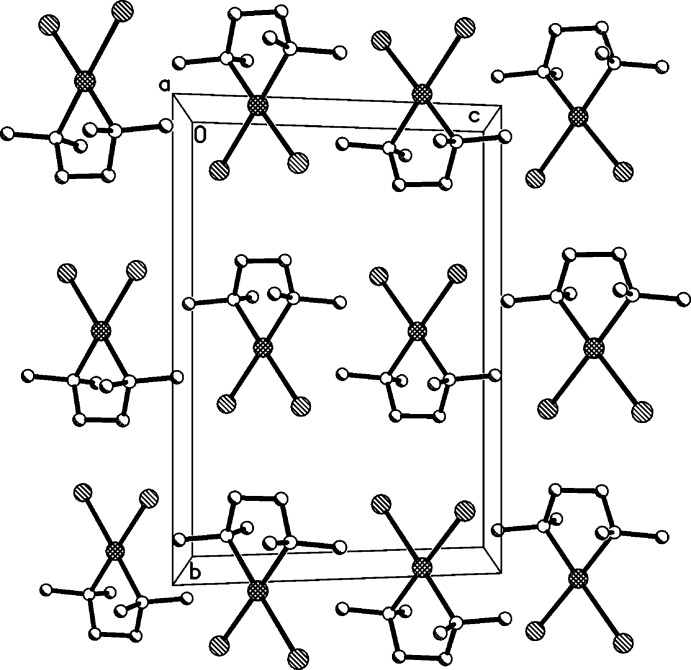
Packing diagram of the title compound view down b axis Hydrogen atoms are omitted.

## supplementary crystallographic information

### Comment

A small amount of the title compound (1) formed when the complex [PdI(C_6_H_4_{NHC(Me)=C{C(O)Me}C(C(CO_2_Me)C=CHCO_2_Me}-2(tmeda)] was heated in toluene with the aim to isomerize it. The insoluble complex 1 was separated by filtration and, single crystals, obtained by the liquid diffusion method using dichloromethane and diethyl ether, were used for struture determintation. The pure compound 1 was prepared as stated in the Experimental section, and crystals grown using the same method and solvents as above were used to confirm the cell dimensions.

Only a few complexes of the type [PdI_2_(N^N)] (N^N = chelating nitrongen donor ligand) have been characterized by their *X* ray crystal structures, namely those with N^N = 4,4'-di-*tert*-butyl-2,2'-bipyridine (Jones *et al.* 2007); 2-((4*S*)-4-isopropyl-4,5-dihydro-1,3-oxazol-2-yl)pyridine (Dodd *et al.* 2006); 1,2-bis(1-pyrrolidino)ethane (Wursche *et al.*, 1999); *N*-2-iodobenzyl-*N*,*N*',*N*'-trimethylethane-1,2-diamine (Alsters *et al.* 1993); 2,2'-bipyridine (Ha 2009); 1,4-dibenzyl-1,4-diazacyclododec-8-ene-6,10-diyne-N1,N4 (Bhattacharyya *et al.* 2009) and 1,10-phenanthroline (Ha 2010). This contrasts with the nearly five hundred crystal structures of dichloro homologous complexes (CCDC, November 2010).

The molecular structure of complex 1 is shown in Fig. 1. The asymmetric unit comprises a half of the molecule as the Pd atom lies on a crystallographic twofold axis. The coordinated ligand atoms and Pd(II) are coplanar within the limits of experimental errors: I1 and N1 are displaced from the least square plane defined by the five atoms by +0.0050 (15) and +0.0062 (19) Å, respectively. The bite of the chelating tmeda ligand displays a N(1)–C(1)–C(1 A)–N(1 A) torsion angle of -56.37(0.60)°. The planes of the two NMe_2_ fragments substend an angle of 12.10(0.08)°.

The Pd–N bonds in the diiodo complex 1 (2.125 (3) Å) are slightly longer than in the homologous dichloro complex [2.053 (3) and 2.073 (3) Å] reflecting the greater *trans* influence of the iodo ligands. The I(1)—Pd(1)—I(1 A) bond angle in 1 (87.907 (18)°) is narrower than the Cl(1)—Pd—Cl(2) one in [PdCl_2_(tmeda)] (Boyle *et al.* 2004) (90.72 (5)°) although the opposite was expected in view of the smaller electronegativity and the bigger size of the iodo ligand compared to chloro. We attribute this fact to the steric hyndrance caused on the iodo ligands by the NMe_2_ groups in the chelating ligand, imposed by the short Pd—N bonds. This is supported by both, the larger Cl—Pd—Cl angle in [PdCl_2_(en)] (Iball *et al.* 1975) (95.3 (3)°) than that in [PdCl_2_(tmeda)] (90.72 (5)°) and on a search at the Cambridge Crystallographic Database (version 5.32, November 2010, updated November 2011) for [Pd*X*_2_(P^P)] (*X* = Cl, I, P^P = phorphorus donor chelating ligands) showing that the I—Pd—I angles are, as expected, wider than the Cl—Pd—Cl ones in homologous complexes, probably because, in this case, the longer Pd—P bond distances keep the phosphorus substituents away enough from the halogen ligands.

### Experimental

*Synthesis.* The pure compound (1) was prepared in 88% yield from [PdCl_2_(tmeda)] and NaI (1:5, in acetone, 3 h at room temperature). The complex was extracted into dichloromethane and precipitated with diethyl ether. *M.p.* 194 °C (decomposition). ^1^H NMR (200 MHz, CDCl_3_): d 2.68 (s, 2 H, CH_2_), 2.96 (s, 6 H, Me). Analysis calcd for C_6_H_16_I_2_N_2_Pd: C, 15.13; H, 3.39; N, 5.88. Found: C, 15.62; H, 3.43; N, 6.03.

### Refinement

Methyl H atoms were identified in difference syntheses, idealized and refined using rigid groups allowed to rotate but not tip, with C—H 0.98 Å, H—C—H 109.5°. Other H atoms were introduced at the calculated positions and refined using a riding model, with methylene C—H 0.99 Å. The *U*_iso_(H) values were set equal to *mU*_eq_(C) of the parent carbons, with *m* = 1.5 for methyls and 1.2 for all other H.

### Crystal data

**Table:**

[PdI_2_(C_6_H_16_N_2_)]	*F*(000) = 872
*M**_r_* = 476.41	*D*_x_ = 2.702 Mg m^−^^3^
Monoclinic, *C*2/*c*	Mo *K*α radiation, λ = 0.71073 Å
Hall symbol: -C 2yc	Cell parameters from 4971 reflections
*a* = 7.9266 (4) Å	θ = 2.8–28.1°
*b* = 14.6911 (7) Å	µ = 6.81 mm^−^^1^
*c* = 10.5309 (5) Å	*T* = 100 K
β = 107.262 (2)°	Needle, red
*V* = 1171.09 (10) Å^3^	0.22 × 0.15 × 0.03 mm
*Z* = 4	

### Data collection

**Table:**

Bruker SMART APEX CCD diffractometer	1351 independent reflections
Radiation source: fine-focus sealed tube	1326 reflections with *I* > 2σ(*I*)
Graphite monochromator	*R*_int_ = 0.020
Detector resolution: 8.26 pixels mm^-1^	θ_max_ = 28.1°, θ_min_ = 2.8°
ω scan	*h* = −10→10
Absorption correction: multi-scan (*SADABS*; Bruker, 2003)	*k* = −18→18
*T*_min_ = 0.581, *T*_max_ = 0.822	*l* = −13→13
6502 measured reflections	

### Refinement

**Table:**

Refinement on *F*^2^	Primary atom site location: structure-invariant direct methods
Least-squares matrix: full	Secondary atom site location: difference Fourier map
*R*[*F*^2^ > 2σ(*F*^2^)] = 0.023	Hydrogen site location: inferred from neighbouring sites
*wR*(*F*^2^) = 0.060	H-atom parameters constrained
*S* = 1.14	*w* = 1/[σ^2^(*F*_o_^2^) + (0.025*P*)^2^ + 10.9315*P*] where *P* = (*F*_o_^2^ + 2*F*_c_^2^)/3
1351 reflections	(Δ/σ)_max_ = 0.001
53 parameters	Δρ_max_ = 1.23 e Å^−^^3^
0 restraints	Δρ_min_ = −0.73 e Å^−^^3^

### Special details

**Table:**

Geometry. All e.s.d.'s (except the e.s.d. in the dihedral angle between two l.s. planes) are estimated using the full covariance matrix. The cell e.s.d.'s are taken into account individually in the estimation of e.s.d.'s in distances, angles and torsion angles; correlations between e.s.d.'s in cell parameters are only used when they are defined by crystal symmetry. An approximate (isotropic) treatment of cell e.s.d.'s is used for estimating e.s.d.'s involving l.s. planes.Least-squares planes (*x*,*y*,*z* in crystal coordinates) and deviations from them (* indicates atom used to define plane)- 0.3294 (0.0368) *x* + 14.6093 (0.0042) *y* + 1.1036 (0.0293) *z* = 6.0620 (0.0112)* 0.0000 (0.0000) N1 * 0.0000 (0.0000) C2 * 0.0000 (0.0000) C3Rms deviation of fitted atoms = 0.00000.3294 (0.0369) *x* + 14.6093 (0.0042) *y* - 1.1036 (0.0293) *z* = 5.8396 (0.0312)Angle to previous plane (with approximate e.s.d.) = 12.10 (0.08)* 0.0000 (0.0000) N1_$1 * 0.0000 (0.0000) C2_$1 * 0.0000 (0.0000) C3_$1Rms deviation of fitted atoms = 0.00005.5219 (0.0048) *x* - 0.0000 (0.0000) *y* - 9.3919 (0.0040) *z* = 0.4130 (0.0034)Angle to previous plane (with approximate e.s.d.) = 84.35 (0.22)* 0.0000 (0.0000) Pd1 * 0.0062 (0.0019) N1 * 0.0050 (0.0015) I1 * -0.0062 (0.0019) N1_$1 * -0.0050 (0.0015) I1_$1Rms deviation of fitted atoms = 0.0050
Refinement. Refinement of *F*^2^ against ALL reflections. The weighted *R*-factor *wR* and goodness of fit *S* are based on *F*^2^, conventional *R*-factors *R* are based on *F*, with *F* set to zero for negative *F*^2^. The threshold expression of *F*^2^ > σ(*F*^2^) is used only for calculating *R*-factors(gt) *etc*. and is not relevant to the choice of reflections for refinement. *R*-factors based on *F*^2^ are statistically about twice as large as those based on *F*, and *R*- factors based on ALL data will be even larger.

### Fractional atomic coordinates and isotropic or equivalent isotropic displacement parameters (Å^2^)

**Table:**

	*x*	*y*	*z*	*U*_iso_*/*U*_eq_	
Pd1	0.5000	0.51798 (3)	0.2500	0.01073 (10)	
I1	0.71154 (4)	0.644568 (18)	0.37384 (3)	0.02349 (10)	
N1	0.3317 (4)	0.4111 (2)	0.1504 (3)	0.0136 (6)	
C1	0.4427 (5)	0.3275 (3)	0.1794 (4)	0.0191 (8)	
H1A	0.5175	0.3246	0.1192	0.023*	
H1B	0.3656	0.2731	0.1632	0.023*	
C2	0.1807 (5)	0.4035 (3)	0.2057 (4)	0.0209 (8)	
H2A	0.1129	0.4603	0.1896	0.031*	
H2B	0.2251	0.3922	0.3016	0.031*	
H2C	0.1045	0.3529	0.1625	0.031*	
C3	0.2605 (6)	0.4205 (3)	0.0038 (4)	0.0216 (8)	
H3A	0.1947	0.3654	−0.0336	0.032*	
H3B	0.3584	0.4291	−0.0341	0.032*	
H3C	0.1816	0.4733	−0.0176	0.032*	

### Atomic displacement parameters (Å^2^)

**Table:**

	*U*^11^	*U*^22^	*U*^33^	*U*^12^	*U*^13^	*U*^23^
Pd1	0.01163 (18)	0.01133 (18)	0.00958 (18)	0.000	0.00366 (13)	0.000
I1	0.02803 (17)	0.02164 (16)	0.01984 (16)	−0.01018 (10)	0.00561 (11)	−0.00239 (10)
N1	0.0124 (14)	0.0169 (15)	0.0119 (14)	−0.0017 (12)	0.0040 (11)	−0.0028 (12)
C1	0.0214 (19)	0.0140 (17)	0.022 (2)	−0.0004 (15)	0.0073 (16)	−0.0038 (15)
C2	0.0145 (18)	0.029 (2)	0.022 (2)	−0.0052 (15)	0.0090 (15)	−0.0034 (17)
C3	0.024 (2)	0.025 (2)	0.0134 (18)	−0.0046 (16)	0.0033 (15)	−0.0043 (16)

### Geometric parameters (Å, º)

**Table:**

Pd1—N1	2.125 (3)	C1—H1A	0.9900
Pd1—N1^i^	2.125 (3)	C1—H1B	0.9900
Pd1—I1	2.5833 (4)	C2—H2A	0.9800
Pd1—I1^i^	2.5833 (4)	C2—H2B	0.9800
N1—C2	1.483 (5)	C2—H2C	0.9800
N1—C3	1.484 (5)	C3—H3A	0.9800
N1—C1	1.488 (5)	C3—H3B	0.9800
C1—C1^i^	1.494 (8)	C3—H3C	0.9800
			
N1—Pd1—N1^i^	84.68 (18)	N1—C1—H1B	109.5
N1—Pd1—I1	178.36 (9)	C1^i^—C1—H1B	109.5
N1^i^—Pd1—I1	93.71 (9)	H1A—C1—H1B	108.1
N1—Pd1—I1^i^	93.71 (9)	N1—C2—H2A	109.5
N1^i^—Pd1—I1^i^	178.36 (9)	N1—C2—H2B	109.5
I1—Pd1—I1^i^	87.906 (18)	H2A—C2—H2B	109.5
C2—N1—C3	108.3 (3)	N1—C2—H2C	109.5
C2—N1—C1	110.7 (3)	H2A—C2—H2C	109.5
C3—N1—C1	108.1 (3)	H2B—C2—H2C	109.5
C2—N1—Pd1	108.9 (2)	N1—C3—H3A	109.5
C3—N1—Pd1	115.7 (2)	N1—C3—H3B	109.5
C1—N1—Pd1	105.2 (2)	H3A—C3—H3B	109.5
N1—C1—C1^i^	110.6 (3)	N1—C3—H3C	109.5
N1—C1—H1A	109.5	H3A—C3—H3C	109.5
C1^i^—C1—H1A	109.5	H3B—C3—H3C	109.5
			
N1^i^—Pd1—N1—C2	104.9 (3)	I1^i^—Pd1—N1—C1	166.5 (2)
I1^i^—Pd1—N1—C2	−74.8 (2)	C2—N1—C1—C1^i^	−77.3 (4)
N1^i^—Pd1—N1—C3	−133.0 (3)	C3—N1—C1—C1^i^	164.2 (4)
I1^i^—Pd1—N1—C3	47.3 (3)	Pd1—N1—C1—C1^i^	40.1 (4)
N1^i^—Pd1—N1—C1	−13.83 (18)	N1—C1—C1^i^—N1^i^	−56.4 (6)

Symmetry code: (i) −*x*+1, *y*, −*z*+1/2.
